# Cost evaluation of cardiovascular magnetic resonance versus coronary angiography for the diagnostic work-up of coronary artery disease: Application of the European Cardiovascular Magnetic Resonance registry data to the German, United Kingdom, Swiss, and United States health care systems

**DOI:** 10.1186/1532-429X-14-35

**Published:** 2012-06-14

**Authors:** Karine Moschetti, Stefano Muzzarelli, Christophe Pinget, Anja Wagner, Günther Pilz, Jean-Blaise Wasserfallen, Jeanette Schulz-Menger, Detle Nothnagel, Torsten Dill, Herbert Frank, Massimo Lombardi, Oliver Bruder, Heiko Mahrholdt, Jürg Schwitter

**Affiliations:** 1 Institute of Health Economics and Management (IEMS), University of Lausanne, Lausanne, Switzerland; 2 Technology Assessment Unit (UET), University Hospital (CHUV), Lausanne, Switzerland; 3 Division of Cardiology and Head of Cardiac MR Centre, University Hospital (CHUV), Lausanne, Switzerland; 4 Department of Cardiology, Hahnemann University Hospital, Drexel University College of Medicine, Philadelphia, USA; 5 Department of Cardiology, Clinic Agatharied, Academic Teaching Hospital, University of Munich, Munich, Germany; 6 WG CARDIAC MRI, Universitätsmedizin Berlin Charité and HELIOS-Klinikum Berlin Buch, Berlin, Germany; 7 Department of Cardiology, Klinikum Ludwigsburg, Ludwigsburg, Germany; 8 Department of Cardiology, Kerckhoff-Klinik, Bad Nauheim, Germany; 9 Landeskrankenhaus Tulln Interne Abteilung, Donauklinikum Alter Zietelweg, Tulln, Austria; 10 C.N.R./Regione Toscana/G. Monasterio Foundation, Pisa, Italy; 11 Department of Cardiology and Angiology, Elisabeth Hospital Essen, Essen, Germany; 12 Department of Cardiology, Robert Bosch Hospital Stuttgart, Stuttgart, Germany

**Keywords:** Cost analysis, Coronary artery disease, Cardiovascular magnetic resonance, Coronary angiography, European Cardiovascular Magnetic Resonance registry

## Abstract

**Background:**

Cardiovascular magnetic resonance (CMR) has favorable characteristics for diagnostic evaluation and risk stratification of patients with known or suspected CAD. CMR utilization in CAD detection is growing fast. However, data on its cost-effectiveness are scarce. The goal of this study is to compare the costs of two strategies for detection of significant coronary artery stenoses in patients with suspected coronary artery disease (CAD): 1) Performing CMR first to assess myocardial ischemia and/or infarct scar before referring positive patients (defined as presence of ischemia and/or infarct scar to coronary angiography (CXA) versus 2) a hypothetical CXA performed in all patients as a single test to detect CAD.

**Methods:**

A subgroup of the European CMR pilot registry was used including 2,717 consecutive patients who underwent stress-CMR. From these patients, 21% were positive for CAD (ischemia and/or infarct scar), 73% negative, and 6% uncertain and underwent additional testing. The diagnostic costs were evaluated using invoicing costs of each test performed. Costs analysis was performed from a health care payer perspective in German, United Kingdom, Swiss, and United States health care settings.

**Results:**

In the public sectors of the German, United Kingdom, and Swiss health care systems, cost savings from the CMR-driven strategy were 50%, 25% and 23%, respectively, versus outpatient CXA. If CXA was carried out as an inpatient procedure, cost savings were 46%, 50% and 48%, respectively. In the United States context, cost savings were 51% when compared with inpatient CXA, but higher for CMR by 8% versus outpatient CXA.

**Conclusion:**

This analysis suggests that from an economic perspective, the use of CMR should be encouraged as a management option for patients with suspected CAD.

## Background

In many countries, cardiovascular disease remains the nation’s most important killer of men and women, causing more than 40% of all deaths in the United Kingdom, 36% in United States and 33% in Switzerland [1-4]. The economic burden of CAD is vitally important also. For instance, the total direct and indirect costs of CAD and stroke were estimated at $ 156 billion in the United States for 2008 and at £ 30.7 billion in the United Kingdom for 2006 [1,5]. In Germany, the total number of invasive coronary angiography (CXA) performed in 2008 accounted for reimbursement costs of over 500 Mio. Euros [6].

Presently, cardiovascular magnetic resonance (CMR) has emerged as a robust, reliable and safe imaging technique for evaluation of myocardial ischemia and infarct scar with high sensitivity and specificity [7-16]. Prognosis in patients with a negative, i.e. normal perfusion-CMR, is excellent with major adverse cardiac event rates as low as 0.3–1.1%/year [17-19]. Thus, CMR is increasingly used in daily routine in many hospitals. While CXA still remains the “gold standard” for evaluation of CAD in many countries, Patel et al. found 62% of elective CXA examinations to be negative for CAD (defined as <50% diameter stenoses) in a large sample of approximately 400,000 US-patients without known CAD [20]. Similarly, in Switzerland in 2010, about two thirds of the CXA tests performed were negative for CAD [21]. In Germany in 2008, only 35% of patients were treated by percutaneous coronary interventions (PCI) and another 7.5% by bypass surgery after CXA [22]. In the United Kingdom, the last available figures on hospitals activities enable to estimate that more than 58% of the performed CXA tests did not lead to invasive cardiac procedures (PCI or CABG) afterwards [1,23]. This suggests that for a considerable number of patients, CXA may not be appropriate. In addition, CXA has some disadvantages such as exposure to radiation, bleeding, and contrast nephropathy.

In a few single centre studies, cost savings were found when using CMR versus conventional CXA strategies for evaluation of chronic and acute ischemic heart disease [24,25]. While such preliminary studies are promising, further investigations are required to better describe the economic impact of CMR utilization. There is general agreement that randomized controlled trials (RCT) are important tools to test hypotheses in a well defined and controlled environment. Once the efficacy of a novel treatment, procedure, or diagnostic test has been demonstrated in a RCT, the question remains whether the same performance will be achieved when applied in a broad community, i.e. in the majority of the health care services. Thus, RCT can answer the question whether a given test or treatment will outperform others in an ideal world, while a registry is the adequate setting to demonstrate in the real-world whether a given test or treatment is still performing as predicted, and consequently, it also appears appropriate to analyze costs generated in a registry environment [26]. In the current study, therefore, a costs analysis was performed in the multicenter European CMR registry [27,28] comparing two strategies for the detection of significant coronary artery stenoses in suspected CAD. CMR was used to assess myocardial ischemia and scar as a first step before referring CAD-positive patients to CXA. Costs were then compared with a second “hypothetical” strategy, where all patients undergo CXA as a single test to detect CAD. This study aimed at assessing the respective costs of these two strategies from a health care payer perspective in the German, United Kingdom, Swiss, and United States health care systems.

## Methods

### Patient population

We used data from the European CMR pilot registry, a multicenter registry including 20 German hospitals and a total of 11,040 consecutive patients with different indications for CMR scans [27]. The most frequent indications were work-up of myocarditis/cardiomyopathies (n = 3,511), risk stratification in suspected CAD/ischemia (n = 3,399), as well as assessment of cardiac muscle viability (n = 1,126). The present analysis focused on patients with clinically suspected CAD who had a stress CMR test. After exclusion of patients with CXA prior to the CMR examination (n = 682), the study population was composed of 2,717 patients (64.4% male gender; mean age 62.4 ± 11.7 years).

The analysis of the CMR examination was done on-site, as was the decision to proceed to revascularization or not, or to add further testing. Centers were instructed to follow established algorithms to assess ischemia, i.e. appearance of ≥1 hypokinetic (or worsening) segment during dobutamine-CMR [11,18] or ≥1 hypoperfused segment(s) during vasodilator induced perfusion-CMR residing in viable (late enhancement negative) tissue [10,13,18,29].

**Figure 1:**
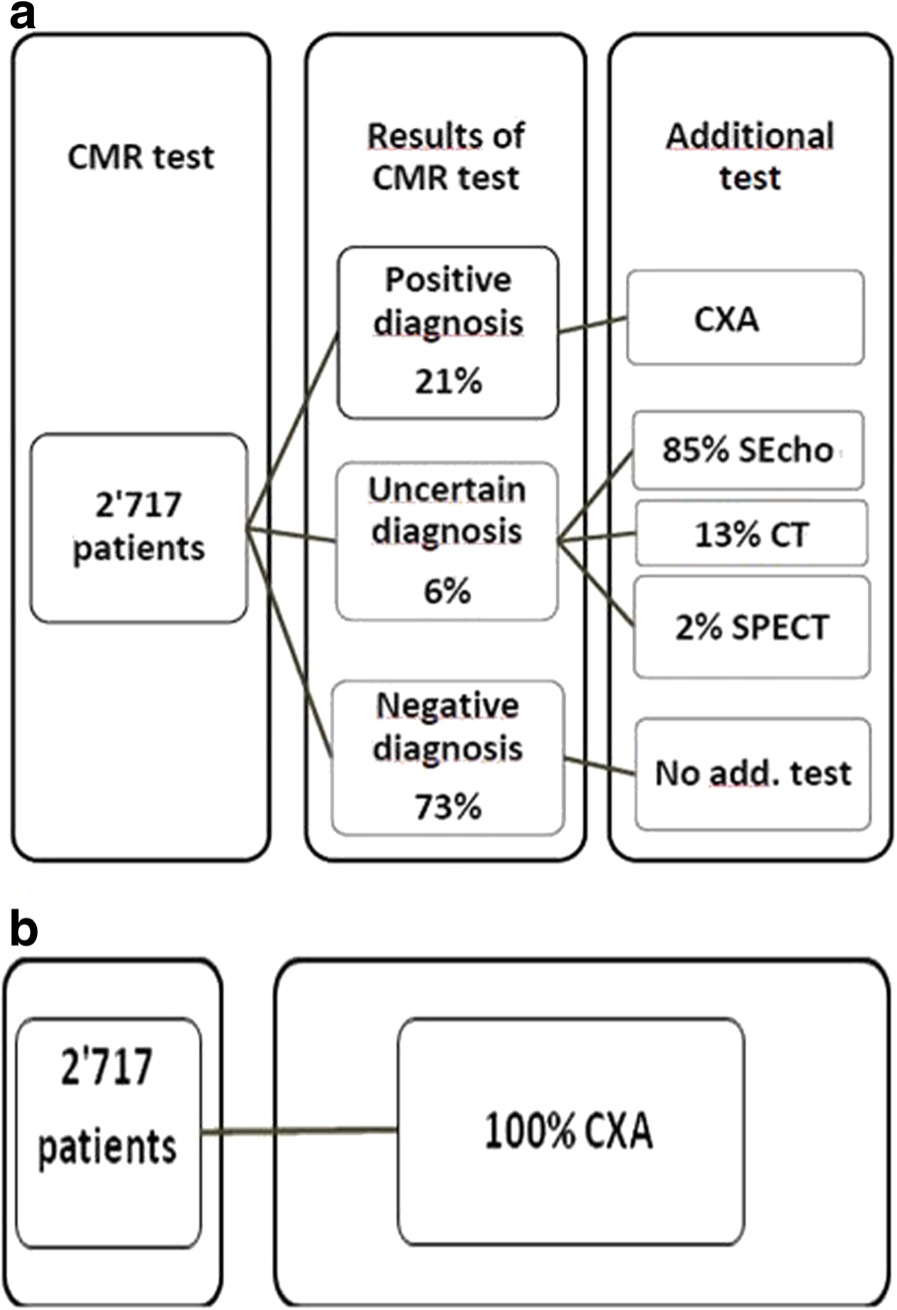
**The two management strategies of the patients.a**) CMR as a “gate keeper” followed by CXA in case of presence of myocardial ischemia **b**) “Hypothetical” CXA in all patients with suspected CAD.

Scar distribution was assessed visually as subendocardial or transmural scar being compatible with CAD [10]. Of the 2,717 patients, 69% underwent adenosine perfusion-CMR and 31% a dobutamine stress-CMR scan. Patients diagnosed positive for ischemia and/or scar by CMR had a CXA. No other test was performed in patients negative for myocardial ischemia and/or scar. Among the study group, the proportion of patients diagnosed positive for CAD was 21%, uncertain 6%, and negative 73% after CMR scan (see Figure1). Those with uncertain diagnosis had additional tests (85% stress echocardiography (SEcho), 13% cardiac CT, 2% SPECT) (see Figure1).

### Costs of the different procedures - Definitions

The analysis was performed from a health care payer perspective using 2011 unit costs data in Euros (€) for Germany, pounds (£) for the United Kingdom, Swiss Francs (CHF) for Switzerland, and American Dollars (US$) for the United States. In the following sections, definitions and descriptions of the health care systems in Germany, the United Kingdom, Switzerland, and the United States are given to explain how the unit costs of tests included in the analysis were derived for each country.

#### Germany

The German health care system relies on public and private health insurers systems. Approximately 90% of the population holds a public insurance policy, the remaining 10% holds a private insurance policy [30]. Costs generated in the private sector were not analyzed in this study.

Public insurance services for outpatient procedures are charged based on a uniform value scale [31]. Inpatient procedures are charged based on the diagnosis related groups (DRG) payment system [32].

In our analyses, SEcho, cardiac CT, and SPECT tests were considered as outpatient tests, while CXA prices were calculated for both, outpatient and inpatient situations. CMR is not yet coded as a specific outpatient examination in the public sector in Germany. In the absence of an outpatient CMR tariff, we opted to use the thoracic MR examination and its related costs as a substitute for the CMR test for the calculations. If a CMR examination is performed in a public hospital, it leads to an admission like all inpatient procedures. However, the patient discharge is done the same day avoiding the hospital stay (e.g. by yielding a normal test result). The CMR test is then reimbursed with a specific code in the public sector (= pre-inpatient test). CMR tests performed as an inpatient procedure in the public sector are covered by the respective DRG.

#### The United Kingdom

The UK National Health Service (NHS) covers all legal residents of the United Kingdom. Primary care services are provided by general practitioners (GP) and hospitals, mainly publicly owned, deliver care to patients referred by GP. Each care event is assigned to a Healthcare Resource Group (HRG) which is a grouping of care events supposed to consume a similar level of resources.

With the exception of the CMR, the costs of the different diagnostic procedures were derived by combining 3 references: the list of procedure codes (OPCS 4.5) with detailed description [33], the national HRGs grouper [34], and the 2009/2010 Reference costs that provide the national average costs for each HRG [23]. For years, CMR (OPCS = U10.3) has been to map to MRI codes with an associated reimbursement (£ 170) that does not match with the costs of CMR. In a transition phase, new procedure codes with higher tariffs/costs are about to be implemented for the CMR in the NHS. We chose to use the new tariff/cost that was suggested by the British Society of Cardiovascular Magnetic Resonance (BSCMR) and the British Society of Cardiovascular Imaging (BSCI).

CMR as well as SEcho, cardiac CT, and SPECT examinations were outpatient tests, while CXA was considered as either an outpatient or inpatient procedure.

#### Switzerland

The Swiss health care system relies on a public health insurance system. Regulated by the Federal Health Insurance Law (LAMal), this system consists of competing private health plans to which each resident in Switzerland is obliged to enroll. These health plans cover the whole range of medical services. Individuals can add private supplementary insurance to fund any additional health care [35].

The costs of all outpatient procedures are coded in the TARMED [36] system and for inpatient procedures a DRG payment system is already implemented in some hospitals in Switzerland, and has been launched in all Swiss hospitals since January 1, 2012. Inpatient unit costs (based on DRGs) were derived for this study from the University Hospital of Lausanne (CHUV), where the DRG system is already implemented.

CMR examinations such as SEcho, cardiac CT, and SPECT were considered as outpatient tests, while costs for CXA were calculated as either outpatient or inpatient procedure.

#### The United States

The costs generated by the different diagnostic procedures in the United States were calculated based on an average national reimbursement as listed in the Current Procedural Terminology codes (CPT) and Clinical Classifications Software version 2011 [37]. For the CXA, CMR, SEcho, SPECT, and cardiac CT performed as outpatient procedures, the respective CPT codes were used. The inpatient CXA cost was calculated by adding the cost of the outpatient CXA procedure and the cost of a hospital stay of one day. The cost of a hospital stay in a « floor bed » for one day was derived from a recent publication reporting the hospitalization costs in the Medicare system [38,39]. All costs were converted to 2011 US dollars using the consumer price index (http://www.bls.gov).

### Costs compilations

The average cost per patient for the two strategies was calculated by using the proportion of patients in the different branches of the diagram (Figure1) and the unit costs of the different tests performed (Table 1). Compilations were performed for the various situations described above.

**Table 1:** Unit costs of the tests performed in Germany, in the United Kingdom, Switzerland, and in the United States

**Unit costs**	**Outpatient in Germany (€)**	**Inpatient in Germany (€)**	**Outpatient in the United Kingdom (£)**	**Inpatient in the United Kingdom (£)**	**Outpatient in Switzerland (CHF)**	**Inpatient in Switzerland (CHF)**	**Outpatient in the United States (US$)**	**Inpatient in the United States (US$)**
CXA	588	1,207	1,055	1,934	2,580	4,638	874	2,652
CMR	164*	393**	558		1,420	/	740	/
SEcho	94	/	213		447	/	303	/
CT	165	/	111		494	/	446	/
SPECT	275	/	406		2,183	/	570	/

* Cost for a thoracic MR examination. ** Cost for MR as a pre-inpatient examination.

As shown in Table 1, large differences exist between the unit costs of the different tests when performed in Germany, the United Kingdom, Switzerland, or the United States. Swiss costs are 1.3 to 3.5 times higher than public United States costs, 2.5 to 4 times higher than the German costs and 1.4 to 3 times higher than the United Kingdom costs for the outpatient tests. In terms of country ranking, this is in accordance with the 2010 International Federation of Health Plans [40] report, which provides actual costs for common medical services across 12 countries. Each health care system has its own organization and specificities, and explaining such differences is beyond the scope of this study. DRG prices for inpatient CXA include the costs of the medical procedures as well as the “hotel costs” associated with the hospital stay. The different components of the total cost were not analyzed here, since the analysis was performed from a health care payer perspective.

## Results

### Cost analysis

**Figure 2:**
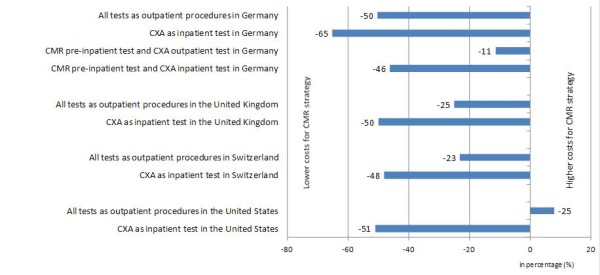
**Percentage of cost variation between CMR strategy and CXA strategy.** When all tests are performed as outpatient procedures in Germany the CMR strategy is 50% less costly than the CXA strategy. By contrast, when all tests are performed as inpatient procedures in the United States, CMR strategy is 8% more costly than the CXA strategy.

Tables 2, 3, 4 and 5 provide the average costs per patient for the two strategies in the various situations in the 4 countries. Figure2 displays the percentage of cost variations for the various situations in the 4 countries.

**Table 2:** Costs related to the two strategies for different situations in Germany

**Costs per patient in different situation in €**	**CMR strategy**	**“hypothetical” CXA strategy**
All tests performed as outpatient procedures in Germany	292*	588
CXA as an inpatient test in Germany	420	1,207
CMR as pre-inpatient test and CXA outpatient test in Germany	521	588
CMR as pre-inpatient test and CXA as an inpatient test in Germany	649	1,207

* Code, i.e. cost for a thoracic MR examination used for calculations.

**Table 3:** Costs related to the two strategies for different situations in the United Kingdom

**Costs per patient in different situation in £**	**CMR strategy**	**“hypothetical” CXA strategy**
All tests performed as outpatient procedures in the United Kingdom	789	1,055
CXA performed as an inpatient procedure in the United Kingdom	970	1,934

**Table 4:** Costs related to the two strategies for different situations in Switzerland

**Costs per patient in different situation in CHF**	**CMR strategy**	**“hypothetical” CXA strategy**
All tests performed as outpatient procedures in Switzerland	1,984	2,580
CXA performed as an inpatient procedure in Switzerland	2,408	4,638

**Table 5:** Costs related to the two strategies for different situations in the United States

**Costs per patient in different situation in US $**	**CMR strategy**	**“hypothetical” CXA strategy**
All tests performed as out-patient procedures in the United States	942	874
CXA performed as an inpatient procedure in the United States	1,308	2,652

#### Situation in Germany

Using costs currently covered by the public health insurance system for the outpatient CMR test, the CMR strategy is 50% less costly than the CXA outpatient strategy (Table 2). When the CMR test is performed by public hospitals as a pre-inpatient test and CXA as an outpatient test, the CMR strategy still remains less costly by 11%.

If CXA is performed as an inpatient procedure (with “hotel costs” included), CMR gate keeping strategy performed as a pre-inpatient test or outpatient test is again less costly by 46% and 65%, respectively.

#### Situation in the United Kingdom

Using the average costs of the procedures calculated across 400 NHS care providers and the CMR costs that will be used in the near future, the CMR strategy is 25% less costly than the CXA outpatient strategy (Table 3). If the CXA procedure is performed as an inpatient test (i.e. spending one night at the hospital), the reduction in costs amounts to almost 51% in favor of the CMR strategy.

#### Situation in Switzerland

When CXA is performed as an outpatient test, CMR strategy costs 23% less than the CXA strategy (Table 4). If the CXA procedure is performed as an inpatient test (i.e. spending one night at the hospital), the reduction in costs amounts to 48% in favor of the CMR strategy.

#### Situation in the United States

In contrast to the previous situations, the CMR strategy costs 8% more than the CXA strategy when all tests are performed as outpatient procedures in the United States (Table 5). Opposite results are found if the CXA procedure is performed as an inpatient test. In this case, the CMR strategy generates a costs reduction of 50% compared with the CXA strategy.

### Breakeven analysis

Based on the proportions given in Figure1, a breakeven analysis was performed in order to assess at which price the CMR strategy would be cost neutral for diagnostic work-up of public outpatients with suspected CAD, i.e. if all tests are performed on an outpatient basis.

For the German system, the results of this analysis suggest that the CMR strategy would be costs saving up to a reimbursement level of Euros 460, while current reimbursement is Euros 164 (code = outpatient thoracic MR) and Euros 393 in public hospitals (pre-inpatient MR). In the United Kingdom, the breakeven analysis found that the CMR strategy would be cost neutral at a reimbursed price of £ 825 while the suggested future price is fixed at £ 558. In Switzerland considering that all tests are performed as outpatient procedures, CMR would be costs saving at a reimbursement level up to CHF 2,015, while the actual price equals CHF 1,420. In the United States, cost savings are achieved by CMR in comparison versus in-patient CXA (Figure2). For this situation, a break even for outpatient CMR is achieved up to a reimbursement for CMR of US$ 2,085, while current reimbursement is set to US$ 740.

## Discussion

While CMR is emerging as a valuable tool to study CAD, data are still rare on costs and cost-effectiveness of this approach versus a conventional invasive CXA strategy to identify patients in need of revascularization. In a recent study conducted in the setting of acute chest pain, Miller et al. found a reduction of hospitalization costs by 23% when using a CMR strategy in an observational unit in the Emergency Department versus an inpatient strategy [25]. Of note, the outcome of the patients was not different for the two approaches, while a large percentage of patients could leave the hospital early when CMR results excluded an acute coronary syndrome [25]. Furthermore, the 1-year costs subsequent to discharge were lower for the CMR patients versus the inpatient admissions [41].

In the current study, we were interested in costs generated by a CMR approach applied in non-emergency situations versus a conventional invasive CXA approach. For this purpose, data from the European CMR pilot registry were used. In this setting, the patient pathway after CMR examination is reported in a routine clinical environment, which is advantageous, if management costs are to be calculated as disease prevalence has a major influence on cost and cost-effectiveness calculations. In the present setting, every positive CMR examination required an additional invasive CXA study to confirm the presence of stenoses and to depict its anatomy, which is a prerequisite to percutaneous or operative treatment of such lesions. Thus, with an increasing prevalence of relevant stenoses in the population undergoing a CMR gatekeeper strategy, its costs will rise. Taking this into account, the utilization of registry data is of great value, as it reflects a real world pre-test prevalence. The data also show a high percentage of approximately 60% of patients being deferred from further testing after a gate keeper CMR examination when performed in a population with a realistic pre-test prevalence. This is in line with other studies that yielded approximately 70% of normal CXA studies [20]. In this context, it should be noted, that CMR is not recommended as a first-line gate keeper test in patients with acute ischemia, e.g. with evidence for acute MI with or without ST elevations.

In addition, it should be recognized that a CMR test yields additional information beyond the presence of myocardial ischemia and scar. CMR allows for quantification of left and right ventricular function, valve function, myocardial viability, and 3D angiography may also be integrated in a CMR examination. In addition, the European CMR registry also yielded strong data underlining the safety of ischemia testing by CMR [42].

### The German health care system

In the German public health care system, the study shows that the utilization of CMR as first non-invasive imaging test in the diagnostic work-up of patients with suspected CAD costs less compared with an invasive CXA strategy. As shown in Figure2, considerable savings could be expected since approximately 865,000 invasive CXA examinations were performed in Germany in 2009, of which 88% were inpatient procedures [6].

### The United Kingdom health care system

Using the CMR as first non-invasive imaging test in the diagnostic work-up of patients with suspected CAD is less costly than using the invasive CXA strategy. Like in Germany and Switzerland (see below), CMR may have a gatekeeper role to invasive examinations in the United Kingdom. A relevant gatekeeper function is highly likely in view of the last available figures in the United Kingdom that tend to show that more than 58% of the performed CXA gave normal results [23].

### The Swiss health care system

For Switzerland, the study shows that the utilization of CMR as the first non-invasive imaging test in the work-up of patients with suspected CAD results in lower costs compared with CXA to all patients and this holds for both, the inpatient and outpatient CXA situation. This suggests a potential role of CMR as a gatekeeper for invasive examinations in Switzerland. The cost saving effect is primarily the result of a reduced number of CXA as approximately 73% of patients in the European CMR registry were deferred from further invasive testing after the CMR examination. In Switzerland the rate of normal CXA studies ranged between 55 to 66% over the last 3 years [21].

### The United States health care system

Data from 2006 demonstrate that approximately two thirds of all cardiac catheterizations were performed on an in-patient basis in the United States [43,44].

Thus, utilization of CMR as a gatekeeper for inpatient CXA could lead to substantial costs reduction in the work-up of patient with suspected CAD.

### Limitations

The proportion of patients undergoing various tests may vary for other populations than the ones we studied. Also, in the United States, the unit costs for the cardiac tests may vary substantially between different geographical regions, and therefore the results are representative for the entire health care system under study, but not for smaller geographical regions.

In this study, the cost analysis was performed from a health care payer perspective. An analysis with a broader perspective would include other costs associated with the diagnostic procedures such as complications and potential future risks induced by CXA radiations for instance, as well as patients’ outcomes. Such an extended analysis could be more relevant to policy makers generally interested in the implications from a societal point of view.

In these registry data, the outcome of the patients deferred from CXA is not known. However, there is increasing evidence for the high prognostic value of CMR ischemia testing. In a cohort of 513 patients with suspected or known CAD, the event rates for cardiac death and non-fatal MI in patients with a negative perfusion-CMR or a negative stress dobutamine-CMR were 0.7% and 1.1%, respectively [18]. In another cohort of 405 patients, these event rates were 0.3% and 1.1% for women and men with a negative perfusion-CMR, respectively (difference not significant) [42]. Short and intermediate term complications of the tests [45] were not considered in the cost analysis. It was not within the scope of this registry to evaluate and thus, to collect the results either of the CXA examinations or of the other alternative tests performed in the patients with inconclusive CMR examinations.

In the present study, it was assumed that the final diagnosis and thus, the decision to revascularize or not, can be reached by coronary angiography. This strategy is still the predominant one in many hospitals. However, there is increasing debate whether the hemodynamic significance of coronary lesions should be assessed e.g. by fractional flow reserve (FFR) to allow for a better clinical decision making [46]. In the current analyses costs were considered for invasive CXA only and no costs for an invasive ischemia testing e.g. by FFR, were added. Thus, the costs for the invasive arm are potentially underestimated. Conversely, for the CMR strategy, information on both ischemia and coronary anatomy was obtained in all ischemia-positive patients.

One might criticize the design which allocated all patients to an invasive procedure in the CXA arm. Whether the pre-test likelihood for having CAD was sufficient to justify an invasive diagnostic procedure in all patients is not known. However, data from Switzerland and the United States show a large proportion of 60%–70% of patients undergoing CXA being negative for CAD, which indicates that in general clinical practice patients with a low to intermediate pre-test likelihood for CAD are indeed sent to CXA. Interestingly, in our study, the negative rate for CAD after the CMR gatekeeper examination was 73%.

One might also criticize that the study compared two strategies for the diagnostic management of CAD without considering other tests. Indeed, other tests such as cardiac CT [47], SEcho, or SPECT when used as a first test may also play an important gate keeper role to CXA. However, the European CMR registry data deal with CMR as a first test and therefore, cannot be used to address other methods as potential gatekeepers.

## Conclusions

This cost analysis performed in a multicenter setting suggests that the development of the use of CMR should be further explored as a management option for patients with suspected CAD. Indeed, it might imply some reductions in the number of CXA examinations and consequently might lead to costs savings together with improved patient safety and comfort. In the context of limited resources in the health care system and the need to permanently optimize their allocation, CMR may lead to a better utilization of resources at the hospital level.

## Abbreviations

CAD, Coronary artery disease; CMR, Cardiovascular magnetic resonance; CT, Cardiac computed tomography; CXA, Coronary angiography; RCT, Randomized controlled trials; SEcho, Stress Echocardiography; SPECT, Single-photon emission tomography; European CMR, European Cardiovascular Magnetic Resonance registry.

## Competing interest

The authors declare that they have no competing interests.

## Authors’ contributions

KM is responsible for the conception and design of the cost analysis, she performed the costs analysis, participated to the data collection and drafted the manuscript. SM participated to data collection and was involved in the results interpretation. CP contributed to the conception and design of the cost analysis. AW provided useful explanations on how the US health care system works and participated in the data acquisition in this context. GP provided precisions on how the German health care system works and actively participated in the acquisition of required data in this context. JBW provided explanations on how the Swiss health care system works. JSM contributed to the collection and assembly of data. DN contributed to the collection and assembly of data. TD contributed to the collection and assembly of data. HF contributed to the collection and assembly of data. ML contributed to the collection and assembly of data. OB contributed to the conception and design of the study, provided detailed explanation on how the German health care system works and actively participated in the acquisition of required data in this context. HM contributed to the conception and design of the study and critically revised the intellectual content of the draft. JS is responsible for the conception and design of the study and the costs analysis, participated to data collection and was involved in the interpretation of the results and drafting the manuscript; he critically revised its intellectual content. In addition, all authors provided helpful comments and relevant suggestions to improve the manuscript and its intellectual content; all authors read and approved the final manuscript.
